# Coping and experience post an adverse birth outcome for fathers: a population-based perspective from India

**DOI:** 10.1186/s12889-025-22823-z

**Published:** 2025-04-25

**Authors:** Moutushi Majumder, G. Anil Kumar, Sarah Binte Ali, Md. Akbar, Sibin George, Siva Prasad Dora, Shuchi Sree Akhouri, Sweta Kumari, Manoj Kumar Singh, Tanmay Mahapatra, Rakhi Dandona, ENHANCE 2020 Team, Moutushi Majumder, G. Anil Kumar, Md. Akbar, Sibin George, Siva Prasad Dora, Rakhi Dandona, Arpita Paul, Arup Kumar Das, Lalit Dandona, Vimal Kumar, Debrupa Bhattacharjee, Dinesh Bhatt

**Affiliations:** 1 Public Health Foundation of India, Saidulajab Extension, New Delhi, 110030 India; 2 Piramal Swasthya Management and Research Institute, Hyderabad, India; 3 Institute for Health Metrics and Evaluation, University of Washington, Seattle, USA

**Keywords:** Bihar, Coping mechanism, India, Newborn death, Stillbirth, Social and cultural practices, Supportive experience

## Abstract

**Introduction:**

We report on the experience and coping mechanism of the fathers post adverse birth outcome from a population-based representative sample in the Indian state of Bihar.

**Methods:**

A state-representative sample of fathers of stillborn babies and babies who died within neonatal period (newborn deaths) born between July 2020 and June 2021 were interviewed. They reported on socio-demography, supportive experience and coping mechanism post birth/death of their baby, and their opinion on if their baby could have been saved. The prevalence of supportive experience, and type and prevalence of coping mechanisms by select socio-demographic characteristics is reported for them, and the prevalence of seeing, holding, and naming of the baby for the fathers of stillborn.

**Results:**

A total of 241 (71.5% participation) and 347 (71.2% participation) fathers of stillborn and of newborn deaths participated, respectively. Being able to talk to someone about their baby was reported by 174 (72.5%; 95% CI: 66.5–77.8) and 264 (77.0%; 95% CI: 72.2–81.1); and having received support to cope with loss by 194 (80.8%; 95% CI: 75.3–85.3) and 264 (77.0%; 95% CI: 72.2–81.1) fathers with stillborn and newborn death, respectively. Majority reported crying as a coping mechanism (70.8%; 95% CI: 64.7–76.3 for stillborn and 75.5%; 95% CI: 70.6–79.8 for newborn deaths), and aggression was the most common negative coping mechanism (29.6%; 95% CI: 24.1–35.7 for stillborn and 28.3%; 95% CI: 23.7–33.3 for newborn death). Majority were of the opinion their baby could have been saved had they gone to a higher-level health facility for delivery or medical attention (63.0% for stillborn and 67.7% for newborn death). Naming, seeing and holding of the stillborn was reported by 5.8%, 83.4% and 55% fathers who were present at the time of delivery, respectively.

**Conclusion:**

This study highlights the need for perinatal bereavement strategies to be inclusive of the fathers along with the mothers and offer insights on formulation of those strategic programs.

**Trial registration:**

Not applicable.

**Supplementary Information:**

The online version contains supplementary material available at 10.1186/s12889-025-22823-z.

## Background

Stillbirths and newborn deaths result in a devastating reality experienced by millions of families worldwide each year, leading to calls for supporting bereaved women and families [1]. While there is a discourse on the impact of a baby’s loss on mothers, it is equally essential to acknowledge the profound and unique experience of paternal grief as well. Fathers, akin to mothers, also form emotional bonds with their unborn child during pregnancy [2, 3], however, they feel side-lined in the event of a perinatal loss [4], and perceive their role mainly as a ‘supporter’ to their partner [5–7].

While there has been considerable emphasis on the involvement of male partners in antenatal and postnatal care services to enhance maternal health outcomes in developing countries [8, 9], literature on the psychological ramifications of pregnancy loss on men themselves is relatively less [7]. In India, limited studies done on bereaved fathers highlight their tendency to avoid discussing or expressing their emotions [10, 11], and consider discussing the loss at home as taboo with the prevailing idea to forget and move on [11]. These traits impact fathers psychologically and necessitate social support but such data are scarce [12]. In this study, we explore the socio-cultural dimensions and experience of fathers’ post-stillbirth and newborn death in a representative sample of fathers in the Indian state of Bihar, which has one of the highest neonatal mortality rate and a significant number of stillbirths in the country [13, 14]. Understanding these aspects will facilitate the development of effective grief interventions inclusive of the fathers to help them better adapt to their loss following a stillbirth or newborn death.

## Methods

### Survey design

Detailed survey design is presented elsewhere [13, 15]. Briefly, Every Newborn Health Assessment and Neonatal Care Evaluation (ENHANCE) 2020 was designed to document change in neonatal mortality rate (NMR) between 2016 and 2020 in the state of Bihar. We estimated a sample of 30,000 livebirths for ENHANCE 2020, assuming a 10% refusal rate and 85% power to detect a reduction of 18% in NMR from 2016 to 2020 at the 95% confidence level (CI). A multi-stage sampling design was used to obtain a representative sample of births from July 2020 and June 2021 among usual resident women aged 15–49 years births from all the 38 districts of Bihar [13]. A total of 267 blocks (50% of the total 534 blocks) were randomly sampled for the survey which included 187 (70%) blocks with only rural population and 80 (30%) blocks with both rural and urban populations to reflect the urban–rural population distribution in the state. Within these 267 blocks, the secondary sampling units (SSUs) were villages in rural areas and urban frame survey blocks in urban areas as defined by the Census 2011 [16]. A total of 1,340 SSUs (941 rural and 399 urban) were sampled in proportion to the number of SSUs in each block, using systematic random sampling.

### Data collection

All households in sampled clusters were enumerated by trained interviewers to document birth outcomes between July 2020 and June 2021 among usual resident women aged 15–49 years and fathers for these birth outcomes. We also documented births between July 2020 and June 2021 for women who had died during or after giving birth to ensure a robust estimation of total births in this population. Following enumeration, all women with stillbirth and neonatal death, and 25% of women with neither between July 2020 and June 2021 selected using systematic random sampling in each SSU were considered eligible for a detailed interview. Fathers of all the sampled births irrespective of the birth outcome were also eligible for a detailed interview.

Relevant to this paper, interviewers trained in study procedures interviewed the fathers of stillborn and newborn death (neonatal death) to document socio-demographic information and if they were present during the delivery, and post-birth experience. Specifically, the fathers were asked if they thought their baby could have been saved, and the reasons for being able or unable to save the baby, opportunities for supportive experiences including being able to talk about their baby with someone, and availability of support or help in coping with the loss. The source of support was documented for those who reported supportive experience. The fathers were read out six statements to understand how they coped with the loss for which they could respond as yes, no, or refused to answer for each statement. The statements included—I kept busy with work, I got involved in physical activities, I engaged in alcohol consumption, I engaged in smoking, I expressed grief by crying, and I expressed grief through aggression. Additionally, the fathers of stillborn were asked if they saw, held or named their baby or if they wanted to.

Data were collected between August 2021 and April 2022. The questionnaire was developed in English and then translated into Hindi (local language), after which these were back-translated into English to ensure the accurate and relevant meaning and intent of the questions in Additional File 1. Pilot testing of the questionnaire was carried out, and modifications were made as necessary. Interviews were captured using the Computer-assisted Personal Interview software on hand-held tablets. At least three to four attempts were made to reach all eligible fathers, including a visit at a later time as per the father’s availability as given by the respondent or other family members, or on holidays for the eligible fathers who were travelling or were at work or not available during the initial round of data collection in the sampled cluster. A total of 20% of interviews were checked in 50% of the 1,340 sampled clusters.

### Analysis

Analysis was undertaken for the fathers who were available in the cluster and were contactable for interviews during the data collection period. Analysis is reported separately for the fathers of stillborns and newborn deaths unless stated otherwise. We present the distribution of fathers with stillbirth and newborn death between July 2020- June 2021 by select socio-demographic characteristics. The distribution of reasons as to why their baby could or could not be saved as reported by the fathers is presented. The distribution of supportive experience and the persons who provided that experience is reported.

We report the prevalence of coping mechanism as positive (I expressed grief by crying, I kept busy with work, and I got involved in physical activities) and negative (I engaged in alcohol consumption, I engaged in smoking, and I expressed grief through aggression) coping mechanism. The reporting of only positive coping, only negative coping, at least one negative coping, and neither is presented. We explored the association of at least one negative and only positive coping mechanisms with supportive experience and select socio-demography variables. Lastly, we present the distribution of whether the fathers of stillborn babies saw, held or named their baby.

We calculated the wealth index quartile to which the father belonged using the standard methods outlined in the National Family Health Survey (NFHS) 4 and 5 as detailed by the Demographic Health Survey program for India [17, 18]. The wealth index quartile one represents the poorest and quartile four the richest. Prevalence is reported with 95% Confidence Interval (CI), Pearson Chi-square test, and z test are reported for significance as relevant. All analyse were performed using STATA 13.1 software (Stata Corp., USA).

## Results

A total of 8,132 fathers were sampled of whom 5,135 (63.2%) fathers were available in the cluster and 2,997 (36.9%) were away from the cluster at the time of survey. Of the 5,135 fathers available in the cluster, 337 were fathers of stillborn and 487 of newborn death. Overall, 3,463 (67.4%) fathers participated in the survey including 241 (71.5%) and 347 (71.2%) fathers of stillborn and newborn deaths, respectively. Distribution of the fathers with adverse birth outcome by select background characteristics is shown in Additional Table 1.

### Possibility to save their baby

**Fig. 1:**
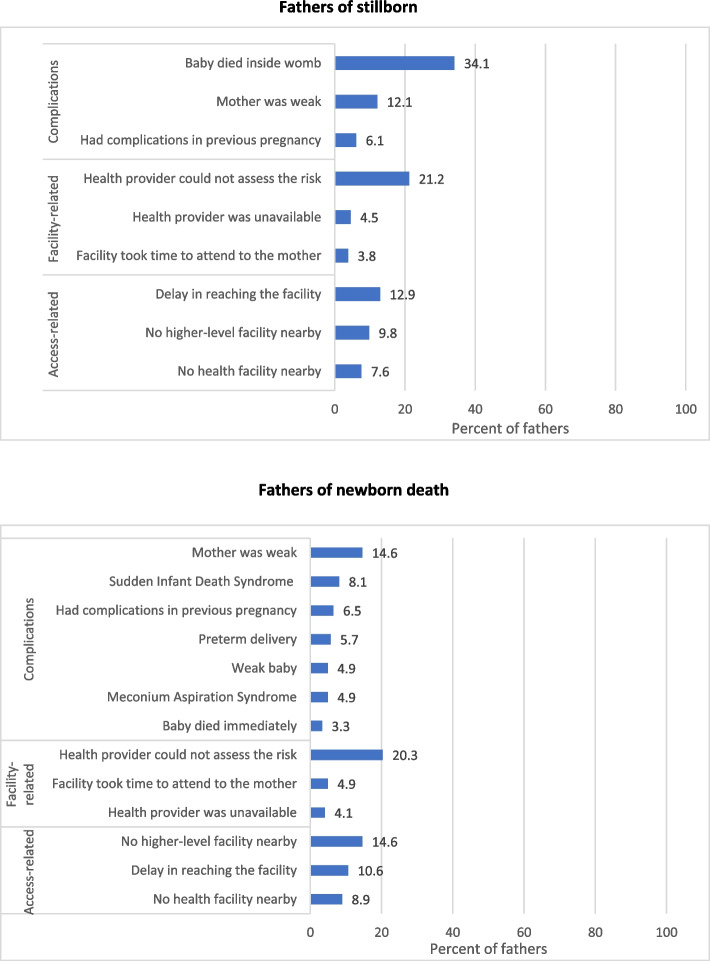
Distribution of the reasons as reported by the fathers of stillborn and newborn deaths who thought there was no possibility of saving their baby. Reasons are not mutually exclusive

A total of 118 (49.2%) of the 241 fathers of stillborn thought that there was no possibility of saving their baby, 108 (45.0%) thought their baby could have been saved, and 14 (5.8%) could not say anything. The major reasons (not mutually exclusive) cited for not being able to save the baby were because the baby died inside the womb (34.1%) and that the health provider could not assess the risk (21.2%) as shown in Fig. 1. Majority of the 241 fathers with stillborn thought that it may have been possible to save their baby if they had gone to a higher-level health facility for delivery or medical treatment (68, 63.0%), followed by if the facility was better equipped to handle complications (29, 26.9%), and if the health provider had not neglected during delivery (11, 10.2%) as shown in Additional Fig. 2.

**Fig. 2:**
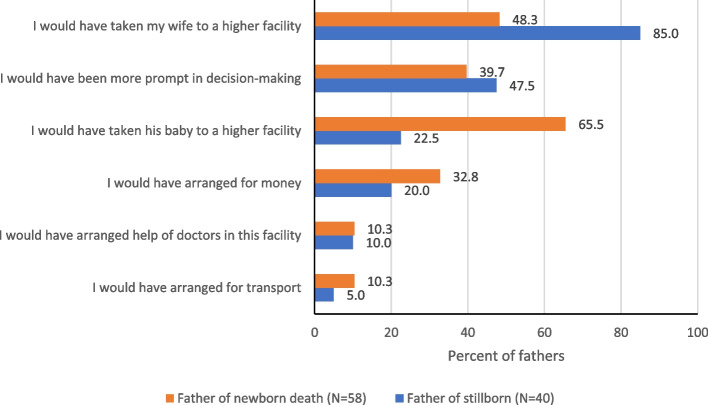
Distribution of the reasons reported by the fathers who thought that their presence during the delivery could have impacted the birth. Reasons are not mutually exclusive

A total of 90 (26.2%) of 347 fathers of newborn deaths thought that there was no possibility of saving their baby, 220 (64.1%) thought their baby could have been saved, and 33 (9.6%) could not say anything. The major reasons reported for not being able to save the baby was that the provider could not assess the risk (20.3%) followed by access-related issues as shown in Fig. 1. Majority of the 347 fathers with newborn death thought it may have been possible to save their baby if they had gone to a higher level of facility for delivery or medical treatment (149, 67.7%), followed by if they had gone to the health facility on time (48, 21.8%), and if the health facility was equipped to handle complications (54, 24.5%) as shown in Additional Fig. 2.

A total of 151 (62.7%) of the 241 fathers with stillborn and 227 (65.4%) of the 347 fathers of newborn deaths were present during the delivery of their baby. Among the 90 (37.3%) and 120 (34.6%) fathers with stillborn and neonatal death who were not present during the delivery, 40 (48.2%) and 58 (50.4%) fathers had the opinion that their presence during the delivery could have resulted in a different birth outcome, respectively. Majority of them reported that they would have taken their wife or baby to a higher-level health facility or would have undertaken more prompt decision making if they were present at the time of delivery (Fig. 2).

### Supportive experience and coping mechanism

**Fig. 3:**
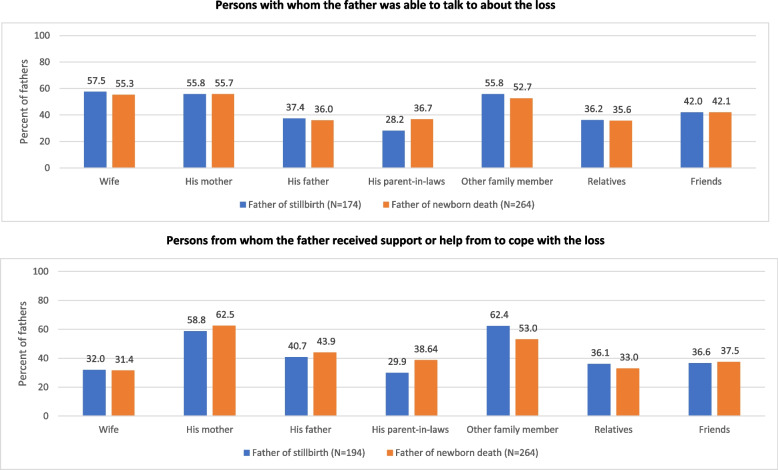
Distribution of persons who provided supportive experience as reported by the fathers with stillbirth and newborn death. Not mutually exclusive

The pattern of supportive experiences was similar for the fathers of stillbirth and newborn death. Being able to talk to someone about their baby was reported by 174 (72.5%; 95% CI 66.5–77.8) of the 241 fathers with stillborn and 264 (77.0%; 95% CI 72.2–81.1) of the 347 fathers with newborn death; having received support or help to cope with the loss was reported by 194 (80.8%; 95% CI 75.3–85.3) of the 241 fathers with stillborn and 264 (77.0%; 95% CI 72.2–81.1) of the 347 fathers with newborn death. The most reported persons with whom they talked about their loss were their wives, their mother, and other family members; whereas the most reported persons from who they received support to cope with the loss were their mother and other family members (Fig. 3). The proportion of fathers reporting wife as a source of support in coping with loss was significantly lower than that being able to talk about their loss with their wives for both the fathers of stillbirth (32.0% vs 57.5%) and newborn death (31.4% vs 55.3%; Z test; *p* < 0.001).

The prevalence of coping mechanisms as reported by the fathers with stillbirth and newborn death is shown in Table 1 (not mutually exclusive). Expressing grief by crying was reported by the majority followed by keeping themselves busy, and getting involved in physical activities. The most common negative coping mechanism reported was expressing grief through aggression by the fathers with stillbirth (29.6%) and newborn death (28.3%), respectively.

**Table 1:** Prevalence of the type of coping mechanisms reported by the fathers of stillborn and newborn deaths between July 2020- June 2021 in the state of Bihar (not mutually exclusive). CI denotes confidence interval

Type of coping	Coping mechanism	Number of fathers with stillbirth^a^ *N* = 241 (% of N; 95% CI)	Number of fathers with newborn death^a^ *N* = 347 (% of N; 95% CI)
Positive	Expressed grief by crying	170 70.8 (64.7–76.3)	259 75.5 (70.6–79.8)
	Kept busy with work	136 56.7 (50.3–62.8)	175 51.0 (45.7–56.3)
	Got involved in physical activities	107 44.5 (38.4–50.9)	149 43.4 (38.3–48.8)
Negative	Expressed grief through aggression	71 29.6 (24.1–35.7)	97 28.3 (23.7–33.3)
	Engaged in smoking	12 5.0 (2.8–8.6)	14 4.1 (2.4–6.8)
	Engaged in alcohol consumption	11 4.6 (2.5–8.1)	21 6.1 (4.0–9.2)

^a^Data not available for 1 and 3 fathers with stillbirth and neonatal death, respectively

A total of 135 (56%), 2 (0.8%), 78 (32.4%), and 26 (10.8%) of the 241 fathers of stillborn reported only positive coping mechanism, only negative coping mechanism, at least one of positive and one negative coping mechanism, and neither, respectively. Among the 347 fathers of newborn death, 197 (56.8%), 5 (1.4%), 109 (31.4%), and 36 (10.4%) reported only positive coping mechanism, only negative coping mechanism, at least one positive and one negative coping mechanism, and neither, respectively.

Considering the 241 fathers of stillborn, those who reported not being able to talk to someone about their loss were significantly more likely to report at least one negative coping mechanism (43.9%) as compared with those who reported were being able to talk to someone about their loss (28.2%; *p* = 0.020). No significant association was seen for the fathers of newborn death.

No significant difference was seen in the reporting of only positive coping mechanism or at least one negative coping mechanism with the age, education or wealth index quartile of the fathers, place of residence, and the sex of the baby for the fathers with stillborn (data not shown). On the other hand, among the 347 fathers with newborn death, the proportion of only positive coping mechanism was significantly higher in rural fathers (59.5%) as compared with urban fathers (45.6%; *p* = 0.038) whereas the latter reported a significantly higher proportion of at least one negative coping mechanism (44.1%) as compared with the former (28.3%; *p* = 0.012). The proportion of at least one negative coping mechanism was significantly higher in the fathers of a boy (36.6%) as compared with fathers of a girl (23.9%; *p* = 0.013).

### Socio-cultural practices with stillborn

A total of 14 (5.8%) of the 241 fathers of stillborn reported that they had named their baby, 132 (55.0%) wanted to name their baby but did not, and 72 (30.0%) did not name their baby because they said that there was no practice of naming a dead baby. Of the 151 fathers who were present at the time of delivery, 126 (83.4%) reported seeing their stillborn baby, 13 (8.6%) did not want to see the baby, 7 (4.6%) reported that their family refused to show, 2 (1.3%) reported that their family suggested it better not to see the dead baby, and data was not available for 3 (2%). Similarly, among the 151 fathers present at the time of delivery, 83 (55%) reported holding their baby, 48 (31.8%) did not want to hold, 13 (8.6%) reported that their family suggested not to hold the baby, 3 (2%) reported that the health provider suggested not to hold the baby, and data was not available for 3 (2%).

## Discussion

To the best of our knowledge, this is the first large-scale population-level study documenting the experience of and coping mechanisms among fathers following an adverse birth outcome. The findings from this study highlight the need for perinatal bereavement strategies to be inclusive of fathers along with mothers and offer insights on the formulation of strategies for intervention.

Three-fourth of the fathers with either stillbirth or newborn death reported being able to talk to someone about their baby loss, and a slightly larger proportion of fathers reported having received support to cope with their baby’s loss. Most of the fathers talked about their loss with their wives; however, dependence on the wives for coping was reported less by the fathers of stillborn as compared with newborn death. The mothers of these men and other family members supported them to cope with their loss. Previous studies have noted that support from the man’s parents can help them alleviate their guilt and sadness about the death of their baby [19, 20]. Studies have reported that fathers of stillborn babies and newborn deaths tend to grieve in isolation as a deliberate choice [21], refraining from sharing their struggles with their partners fearing that they might amplify their partner's grief [22], which often leads to their grief being overlooked [7, 22]. Consequently, they may choose not to express pain or seek the essentially needed psychosocial support [23]. It is recommended that couples should engage in healthy dyadic coping and interactional processes to cope with loss effectively [24], and hence it is vital to formulate perinatal bereavement strategies to be inclusive of fathers.

Notably, majority of the fathers in this study reported positive type of coping mechanism, with crying for coping reported by most followed by keeping busy with work and doing more physical activities. It is noted in the previous studies that fathers often adopt instrumental grieving style which focusses on routine distractions, including being busy and involvement in physical activities [25], rather than emotional expression such as crying [5, 25–27]. A few studies have reported that an outward emotional grief expression such as crying [5] can be beneficial for one's well-being [28]. We believe that crying was reported by a higher proportion of fathers in our study because crying was read out by the interviewer as one of the possible response options under the coping mechanisms. In the background of gendered outlook to crying [29, 30], this explicitness could have resulted in normalisation of crying for them and encouraged them to respond more openly about it. This is an important finding highlighting that we need to re-think our culturally conditioned approach to men crying [31], and normalise it as a healthy emotional response to challenging situations including loss of a baby while considering perinatal bereavement strategies for fathers.

This study found that nearly 30% of the fathers reported coping through aggression, in particular fathers of stillborn babies. Potential coping reported by fathers following the loss of a baby includes a tendency to resort to avoidance behaviour by substance abuse [6, 7, 32, 33], and aggression [25]. Aggression is a maladaptive coping strategy [25, 34], and a possible explanation is that grieving fathers tend to use aggression to avoid feelings of fear brought on by the loss of the baby [35]. Notably, the avoidance and coping behaviour could result in work challenges and financial indebtedness [33, 36], impaired social framework for families [37], with both physical and emotional relationships impacted [10, 25], along with prolonged grief [7]. Fathers need healthy coping strategies and support networks propelled through counselling and support services to address emotional distress [21, 25, 32].

Coping after newborn death was found to be better among fathers in rural areas as compared with their urban counterparts in our study. This difference may be attributed to a more robust support system in the rural communities [38–40]. Families and friends play a central role in offering emotional and practical support following the death of a baby [41]. Urban environments often have a higher social isolation and stress level, which can hinder individuals’ coping mechanism because of poor support system [10, 39, 40]. Addressing social isolation and strengthening community support networks in urban areas could help improve coping outcomes for the fathers.

Among the fathers who were not present during delivery of their baby, one in two fathers of stillborn and newborn death believed that their presence during delivery could have possibly altered the birth outcome. The bereaved fathers in this study cited reasons both related to healthcare providers'actions or self-blame that could have influenced the birth outcome of their baby [42] Other studies indicate that many fathers believe stillbirths are preventable, often attributing them to biomedical shortcomings [43], leading to feelings of anger and dissatisfaction toward hospitals or care providers [10]. Broadly, the sense of regret could be a reflection of guilt [6, 44, 45], loss of control [44, 46], self-blame [46], or a desire to have been more involved in decision-making processes during childbirth. The feeling of helplessness and losing control has been reported for the fathers [10, 44, 47], but the perspective of fathers who were not present during the delivery of the baby has not been reported previously to the best of our knowledge.

Globally, interventions involving seeing and holding the baby have been shown to improve the well-being of the bereaved parents [48, 49] and spending time with the stillborn child is considered a ritual in some cultures [50–52]. Despite the feeling of grief and loss, mourning for a stillborn is not encouraged in India by both the family and healthcare providers [52, 53]. However, majority of the fathers in this study who were present during the delivery reported seeing their stillborn baby. This could be because the mothers are discouraged from seeing their stillborn baby in an Indian setting, and the fathers are expected to take care of the routine administrative procedures and manage the baby post-delivery [54]. This could be an opportunity for the healthcare providers to help the fathers cope with their grief and navigate their roles in the aftermath of stillbirth as a bereaved parent sees their baby for the first time [55–57].

The insights from this study indicate that focused attention on fathers to cope with adverse birth outcome is essential, which will require an open and effective communication, and compassionate and respectful care [58]. Mentoring healthcare providers is a common strategy to improve service delivery in maternal and newborn care, which should also include capacity building for counselling of the fathers in addition to the mothers on positive coping and aligning with the needs of the bereaved parents.

There are several strengths of this study. First, it is a population-level reasonably large sample of fathers’ representative of the adverse birth outcomes in the state as compared with small convenience sample or hospital-based sample. Second, it has captured experiences of both the fathers of stillborn and newborn death using the same methodology, thereby, allowing for a more robust comparison of the experiences between the two. Third, it has documented experiences of the fathers irrespective of their presence at the time of delivery. There is also a limitation in terms of participation of the fathers that needs to be considered, however, this is not unique to this survey. Additionally, given the observational nature of the study, any determined association should not be interpreted as causal.

## Conclusion

In conclusion, the findings from this study highlight the need for perinatal bereavement strategies to be inclusive of fathers along with the mothers, and offer insights on formulation of such strategies.

## Supplementary Information

Supplementary Material 1.

Supplementary Material 2.

## Data Availability

All data and materials relevant to the study are included in the article or uploaded as supplementary information.

## Abbreviations

ENHANCE 2020: Every Newborn Health Assessment and Neonatal Care Evaluation2020
NMR: Neonatal Mortality Rate
CI: Confidence Interval
