# Supplementary material for: Coping and experience post an adverse birth outcome for fathers: a population-based perspective from India
Source: BMC Public Health. 2025 Apr 25;25:1542. doi: 10.1186/s12889-025-22823-z (PMC12023662; doi:10.1186/s12889-025-22823-z)
Supplement: Supplementary file 1 — Supplementary Material 1. [file 12889_2025_22823_MOESM1_ESM.docx]

**Coping and experience post an adverse birth outcome for fathers:**

**a population-based perspective from India**

Moutushi Majumder, G Anil Kumar, Sarah Binte Ali, Md. Akbar, Sibin George, Siva Prasad Dora, Shuchi Sree Akhouri, Sweta Kumari, Manoj Kumar Singh, Tanmay Mahapatra, Rakhi Dandona, ENHANCE 2020 Team

Correspondence to: Prof. Rakhi Dandona, Public Health Foundation of India, Saidulajab Extension, New Delhi-110030, India; [rakhi.dandona@phfi.org](mailto:rakhi.dandona@phfi.org)

Additional Table 1. Distribution of the fathers of stillborn and newborn death for births between July 2020- June 2021 in the state of Bihar by background characteristics.

| **Background characteristics** | **Number of fathers with stillbirth**  **N=241 (%)** | **Number of fathers with newborn death**  **N=347 (%)** |
| --- | --- | --- |
| **Father’s age**^§^ |  |  |
| 18- 24 years | 55 (22.8) | 81 (23.3) |
| 25-29 years | 75 (31.1) | 136 (39.2) |
| 30-34 years | 55 (22.8) | 65 (18.7) |
| 35-39 years | 28 (11.6) | 40 (11.5) |
| 40 years & above | 28 (11.6) | 25 (7.2) |
| **Father’s education**^¥^ |  |  |
| No education | 61 (25.3) | 85 (24.5) |
| Class 1-5 | 54 (22.4) | 81 (23.3) |
| Class 6-12 | 99 (41.1) | 146 (42.1) |
| More than class 12 | 27 (11.2) | 35 (10.1) |
| **Wealth index quartile***^¶^ |  |  |
| 1 | 58 (24.2) | 96 (27.7) |
| 2 | 54 (22.5) | 76 (21.9) |
| 3 | 56 (23.3) | 93 (26.9) |
| 4 | 72 (30.0) | 81 (23.4) |
| **Place of residence**^€^ |  |  |
| Urban | 56 (23.2) | 68 (19.6) |
| Rural | 185 (76.8) | 279 (80.4) |

* Data not available for 1 father with stillbirth and newborn death. ^§^ Chi-square test for significance: p=0.015 for stillbirth and p=0.071 for newborn death.

^¥^ Chi-square test for significance: p=0.030 for stillbirth and p=0.003 for newborn death.
^¶^ Chi-square test for significance: p=0.878 for stillbirth and p=0.023 for newborn death.

^€^ Chi-square test for significance: p=0.784 for stillbirth and p=0.169 for newborn death.

Additional Figure 2. Distribution of the reasons as to why the fathers of stillborn and newborn deaths thought there was a possibility of saving their baby. Reasons are not mutually exclusive.
