# Supplementary material for: Coping and experience post an adverse birth outcome for fathers: a population-based perspective from India
Source: BMC Public Health. 2025 Apr 25;25:1542. doi: 10.1186/s12889-025-22823-z (PMC12023662; doi:10.1186/s12889-025-22823-z)
Supplement: Supplementary file 2 — Supplementary Material 2. [file 12889_2025_22823_MOESM2_ESM.docx]

| **F. Father’s Tool** | | | |
| --- | --- | --- | --- |
|  | **General section** | | |
| F. | Interviewer ID  Supervisor ID  District ID: Block ID:  Cluster ID:  Structure ID:  HH ID:  Focal child ID:  Focal child father ID: | \| \| \|  \| \| \|  \| \| \|  \| \| \| \|  \| \| \| \| \|  \| \| \| \|  \| \| \|  \| \| \|  \| \| \| | |
| F1. | Name of the focal child | Name ________________ |  |
| F1a. | Result of 1^st^ /2^nd^ /3^rd^ visit | Available for interview  Refused  Not available at this time but in town  Not in town (travel)  Temporarily migrated  Permanently migrated | 1  2  3  4  5  6 |
| ***If F1a=1, go to F2***  ***If F1a=2 or 6, END the survey*** | | | |
| F1b. | Please provide when the respondent is likely to be available. | Date  Time | \|_\|\|_\|\|_\|\|_\| |
|  | **END INTERVIEW** | | |
| F2. | What is your name? | Name _______________ |  |
| F3. | INTERVIEWER - Did the respondent give consent for the interview? | Yes No | 1 0 |
| **If F3=0, END the survey** | | | |
| F4. | Please tell me your age. | Age in completed years | \| \| \| |
| F5. | How old were you when you first got married? | Age in completed years | \| \| \| |
| F6. | What is the highest standard or class you have completed? | Never been to school  Non-formal education  Primary (class 1-5)  Upper primary (class 6-7)  High (class 8-10)  Senior secondary (class 11-12)  College/diploma or higher | 1  2  3  4  5  6  7 |
| F7. | What is your main occupation? | Unemployed Homemaker Service/salaried worker  Agricultural labour Cultivator Farmer other than cultivator Business/self-employed  Labour Other (specify) | 1  2  3  4  5  6  7  8  88 |
| **Migration** | | | |
| F8. | Do you stay here all the time or migrate for some part of the year for work? | No, stay here all the time Migrate elsewhere | 0 1 |
| **If F8=0, go to F12** | | | |
| F9. | Where do you migrate to– within or outside Bihar? | Within Bihar Outside of Bihar | 1 2 |
| F10. | In which month did you return home now (this trip) in 2021? | Month Not applicable | \|_\|\|_\| 99 |
| **If F10=99, go to F12** | | | |
| F11. | Had you planned to return home at this time or you came back due to Covid-19? | Had planned to return this time Due to Covid-19 | 1 2 |
| **Father’s opinion and perceptions** | | | |
| **Please explain to the respondent: Now, I am going to ask you questions about your thoughts on and understanding of the ANC services in general. These are not about what your wife did during her pregnancy.** | | | |
| F12. | In your opinion, is antenatal check-up needed during the pregnancy? | Yes No Cannot say | 1 0 95 |
| **If F12=0 or 95, go to F14** | | | |
| F13. | What are the reasons why check-up during pregnancy is needed?    **PROBE CODE ALL THAT APPLY** | To manage pregnancy well  To prepare for delivery  For better health of the baby  For better health of the mother To avoid complications during pregnancy  To receive on advice on delivery  To receive advice on new born care Other (specify)________ | 1 2 3 4  5  6  7  88 |
| **Go to F15** | | | |
| F14. | What are the reasons why you think that the check-up during pregnancy is not needed?  **PROBE**  **CODE ALL THAT APPLY** | Most pregnancies are normal  Elders at home know what is needed  It is needed only if a risk is suspected  No specific reason  Other (specify)________ | 1 2 3  4 88 |
| F15. | Do you know what check-up is usually done for a pregnant woman during antenatal check-up?  **CODE ALL THAT APPLY** | Do not know Weight check  Blood pressure check  Abdomen check Blood sample is taken  Urine sample is taken Tetanus injection given Counselling done Referral given for complication  Ultrasound scan  Other (specify)______  Refused to answer | 98 1 2 3 4 5 6 7 8 9  88  97 |
| F16. | Do you know what tablets/medicines are provided to a pregnant woman during antenatal check-up?  **CODE ALL THAT APPLY** | Do not know  Iron and Folic Acid tablets  Calcium tablets  Other (specify)____ | 98 1 2  88 |
| F17. | Do you think that men in general should be more involved in the pregnancy than what is usually seen? If yes, how?  **CODE ALL THAT APPLY** | No  Yes, planning the pregnancy  Yes, planning the place of delivery  Yes, accompanying for ANC visits  Yes, accompanying for delivery  Yes, emotional/moral support  Other (specify)_____ Cannot say | 0 1  2  3  4  5  88  95 |
| **INFORMATION ABOUT FOCAL CHILD PREGNANCY**  **Please explain to the respondent: Now, I am going to ask you questions about the ANC services that your wife may have sought for pregnancy with [CHILD’s NAME].** | | | |
| F18. | When your wife got pregnant with (focal child), did both of you want a baby at that time? | Only I wanted, she did not want  She wanted, I did not want  Both wanted  Both did not want  Cannot say Refused to answer | 1  2  3  4 95 97 |
| F19. | Did your wife go for routine antenatal check-up during the pregnancy with this (focal child name)? This could be even one ANC visit. | No  Yes  Do not know | 0 1  98 |
| **If F19=1, go to F21** | | | |
| F20. | What were the reasons for your wife not going for ANC check-up?  PROBE  **CODE ALL THAT APPLY** | **Individual** ANC not necessary  ANC not customary Had previous children without ANC  Financial constraints  **Family related**  Family responsibilities kept her busy  Family did not give permission  No one to accompany her She went to her parent’s house for delivery  **Accessibility/availability/quality of services** Fear of Covid-19 Facility staff asked to come later due to lockdown Too far/ no transportation  Could not afford transport  Unhygienic practices in public facilities  Unavailability of transportation due to Covid-19  Do not know  Other (specify)_________ | 1  2  3  4  5  6  7  8  9  10  11  12  13  14  98  88 |
| **Go to F24** | | | |
| F21. | How many times did your wife go for antenatal check-up for (focal child name)? | Number of times Don’t know/recall | \|_\|\|_\| 98 |
| F22. | How many times, did you accompany your wife ANC visits for (focal child name)? | Number of times  Not applicable | \|_\|\|_\|  99 |
| **If F22>0, go to F24** | | | |
| F23. | Please tell me why you did not go with your wife for ANC visits for (focal child name)?  **PROBE**  **CODE ALL THAT APPLY** | Men do not typically accompany for ANC  Was not at home  Went somewhere for work  Was busy on the day Did not know I could also go  No apparent reasons My wife was in her parental home Other (specify)__________  Refused to answer | 1  2 3 4 5  6  7  88  97 |
| F24. | Did any health provider counsel you for birth preparedness during pregnancy with (focal child name)?  If yes, who?  **CODE ALL THAT APPLY** | Not counselled  Doctor ASHA ANM AWW Other (specify)__________ | 0  1 2 3 4 88 |
| F25. | During your wife’s pregnancy with (focal child name), did ASHA, AWW, or ANM talk with you or your wife or your mother about how to prepare for an emergency that could arise during pregnancy or delivery?  **CODE ALL THAT APPLY** | Yes, talked with my wife  Yes, talked with me  Yes, talked with my mother  Not talked with anyone  Do not know | 1  2  3  4  98 |
| **If F25>3, go to F27** | | | |
| F26. | Did they talk to any one of you about identifying a facility to go to in case of an emergency? | Yes  No  Do not remember | 1 0  97 |
| F27. | Did you identify any health facility in case of an emergency?  **CODE ONE ONLY** | Not identified PHC CHC SDH APHC DH  MCH Private facility Private clinic Other (specify)_____ | 0 1 2 3 4 5 6 7  8 88 |
| F28. | During your wife’s pregnancy with (focal child name), what concerns did you have about your baby’s health?  **CODE ALL THAT APPLY** | No concerns I was worried about baby's movements  People smoking around my wife  Baby's growth and health  About my wife getting infected with Covid-19  About the baby getting infected with Covid-19 Baby may die if not delivered at a facility  Other (specify)______________ | 0 1 2 3 4  5  6  88 |
| F29. | Are you aware of any government schemes that provide money to mothers for delivery in a facility, such as Rs. 1,400 schemes etc.?  **EXPLAIN THE SCHEMES FOR CLARITY.**  **PROBE; CODE ALL THAT APPLY** | No  Yes, JSY (1,400/1,000)  Yes, JSSK  Yes, SVA/BVA  Yes, aware of others, specify  _________________________ | 0  1  2  3  88 |
| F30. | Did (focal child name) delivery happen at home, at a health facility or on route to facility? | At home  Health facility On route to facility | 1 2 3 |
| **If F30>1, go to F33** | | | |
| F31. | Was it a planned home delivery? | No  Yes  Don’t know | 0  1 98 |
| **If F31=0, go to F38** | | | |
| F32. | Why was delivery of (focal child name) planned to be at home?  **PROBE**  **CODE ALL THAT APPLY** | Had delivered at home earlier I feel more comfortable Family decided Have seen others delivering at home  Did not think of planning  Health care provider denied due to Covid-19 Called government ambulance but they denied duty due to Covid-19  Due to financial costs  Due to floods  Due to road under construction  No transportation was available Other (specify)_______ Refused to answer | 1 2 3 4 5 6  7  8  9  10  11  88 97 |
| **Go to F38** | | | |
| F33. | Was it a planned facility delivery? | No  Yes | 0 1 |
| F34. | Which type of facility was the baby delivered at – public or private? | Public facility  Private facility  On route | 1  2  3 |
| **If F34=1, go to F35**  **If F34=2, go to F36**  **If F34=3, go to F37** | | | |
| F35. | Why did you plan for this delivery to be at a public health facility?  **PROBE**  **CODE ALL THAT APPLY** | Less expensive Better quality of service Have done it for previous child Wife had complications  Was asked so by FLW  It provides incentive for delivery No other health facility nearby  Proximity of the health facility  Dissatisfied with previous delivery at private facility  Was suggested by family/relatives Other (specify)______ Don’t know | 1 2  3 4 5  6 7  8  9  10  88 98 |
| **Go to F37** | | | |
| F36. | Why did you plan for this delivery to be at a private health facility?  **PROBE**  **CODE ALL THAT APPLY** | **Quality of service** Better quality of service Not happy with the previous delivery at public sector  Better hygiene standards **Advice related** Was suggested by the public sector staff Family suggested **Emergency** Wife had complications  C-section was needed **Accessibility** Private facility was nearby Less transportation cost  Motivated by the govt insurance scheme Other (specify)______  Don’t know | 1 2  3  4 5  6 7  8  9  10  88 98 |
| F37. | Did you receive any financial assistance for delivery from any government program? | Yes  No Don’t know | 1  0 98 |
| **Interviewer: Now, I am going to ask you about your experience related to delivery of the (focal child’s name).** | | | |
| F38. | At any time when your wife was pregnant with (focal child name), did any health provider or health worker ever tell you about any signs of pregnancy complications? | No Yes Do not know/recall | 0 1 96 |
| **If F38=0 or 96, go to F40** | | | |
| F39. | What complications were informed by the health provider or health worker?   **PROBE**  **CODE ALL THAT APPLY** | Explained but do not remember Convulsion  High blood pressure  Cord around baby’s neck Diabetes Malaria Swelling of hands, body or face Gum related disease Fever Vaginal bleeding Severe abdominal pain Prolonged labour  Breech position of the baby  Low fetal heartbeat/movement  Small size of the baby  Other (specify)___________ | 0 1 2 3 4 5 6 7  8  9 10 11  12  13  14  88 |
| F40. | Were you present with your wife at the time of delivery of (focal child name)? | No  Yes, in the delivery room  Yes, outside the delivery room Other (specify)___________ | 0 1  2 88 |
| **If F40>0, go to F43** | | | |
| F41. | Why were you not present during the delivery of (focal child name)?  **PROBE**  **CODE ALL THAT APPLY** | I was not in town My wife was at her parental place  Delivery happened suddenly and I was not around  I was at work  She had other members with her  Was not allowed due to Covid-19 restrictions  Did not think it was necessary Other (specify)_____________ | 1 2 3  4 5 6  7  88 |
| F42. | Who supported for care of mother/child during the delivery?  **CODE ALL THAT APPLY** | Wife herself My parents Her parents Other family members Friends ASHA AWW  ANM  Dai Other (specify)__________ | 1 2 3 4 5 6 7  8  9  88 |
| F43. | What kind of worries did you have during the delivery?  **PROBE**  **CODE ALL THAT APPLY** | No worries  About the pregnancy outcome  Fear of complications Fear of Covid-19 infection  Fear of unavailability of transportation  Lack of support of the family members Lack of financial support  Fear of my wife’s life Other (specify)_____________ | 0  1 2 3 4 5 6  7  88 |
| F44. | Were any challenges experienced during the delivery of (focal child name)? | Yes No  Cannot say | 1 0  95 |
| **If F44=0 or 95, go to F46** | | | |
| F45. | What were the challenges that were faced during the delivery of (focal child name)?  **PROBE**  **CODE ALL THAT APPLY** | No FLW was available for delivery Doctor/nurse denied service Health staff referred elsewhere Multiple referrals from facility to facility Facility staff asked to come later  No beds were available  Wife had complication  Baby died in the stomach  Baby was upside down  No TBA available on time  Issue with the non-availability of transport  Other (specify)________ | 1 2 3 4 5 6 7  8  9  10  11  88 |
| F46. | Interviewer: Please specify the status of the focal child of this father | Stillbirth  Neonatal death on day 0  Neonatal death after day 0  Child alive | 1 2 3  4 |
| **If F46 =4 go to F79** | | | |
| **Fathers with adverse pregnancy outcome** | | | |
| F47. | In your opinion, do you think that it was possible to save your baby? | No  Yes  Cannot say | 0 1 95 |
| **If F47=0 or 95, go to F49** | | | |
| F48. | Why do you think that it was possible to save your baby?  **PROBE**  **CODE ALL THAT APPLY** | **Individual level** Should have gone to a health facility on time  Should have gone to a higher-level health facility  **Facility related**  Had the health provider not neglected  Had the health provider not sent back  Had the health provider not referred  Had the health facility was equipped to handle complications  Had the provider not delayed the C-section  Other (specify)________ | 1  2  3  4  5  6  7  88 |
| **Go to F50** | | | |
| F49. | Why do you think that it was not possible to save your baby?  **PROBE**  **CODE ALL THAT APPLY** | **Individual level**  Mother was weak  Had complications in previous pregnancy  Provider could not assess the risk  **Access related**  No health facility nearby  No higher-level facility nearby  Health provider was unavailable  Delay in reaching the facility  Facility took time to attend to the mother  Other (specify)_______ | 1  2  3  4  5  6  7  8  88 |
| F50. | **INTERVIEWER** – Was the respondent present during the delivery? | No  Yes | 0 1 |
| **If F50=1, go to F53** | | | |
| F51. | Do you think that the outcome would have been different if you were present during the delivery? | Yes No Don’t know | 1 0 98 |
| **If F51=0 or 98, go to F53** | | | |
| F52. | How do you think that your presence during the time of delivery would have impacted this (focal child) birth?  **PROBE**  **CODE ALL THAT APPLY** | Would have taken my wife to a higher facility Would have taken my baby to a higher facility More prompt decision making  I would have arranged for money  I would have arranged for transport  Would have arranged help of doctors in this facility  Would have preferred C-section  Would have spoken to the provider/hospital authority  Other (specify)_______ | 1  2  3  4  5  6  7  8  88 |
| F53. | What were you told by the health provider when your baby died/about your stillborn baby?  **PROBE**  **CODE ALL THAT APPLY** | Nothing was told Baby had breathing issue  Baby was very weak Baby had cord around neck Baby had congenital issue Mother and baby were weak Baby was dead inside  Baby had infection Other (specify)__________ | 0 1 2 3 4 5  6  7 88 |
| F54. | Did you get any support from the health provider after your baby died/about your stillborn baby?  **CODE ALL THAT APPLY** | No support  Counselling done  He/she felt sorry for our loss Suggested place of cremation/burial Other (specify)_____________ | 0 1 2 3 88 |
| F55. | What kind of support would you have liked from the health provider?  **PROBE**  **CODE ALL THAT APPLY** | Cannot say Counselling  Show of sympathy  Talk nicely with us Support in cremating/burying the baby  Other (specify)_________ | 0 1 2  3  4  88 |
| **If F46=3, go to F61** | | | |
| F56. | How long after the delivery, was the baby handed over to you/family? | Hour  Minutes | \| \|  \| \| \| |
| F57. | Did you see your baby?  **PROBE**  **CODE ALL THAT APPLY** | Yes  No, I did not want to see  Health provider refused to show  Health provider suggested not to see  Family refused to show  Family suggested better not to see  I was not present during the delivery  Other (specify)______ | 1  2  3  4  5  6  7  88 |
| F58. | Did you hold your baby? | Yes  No, I did not want to hold  Health provider suggested not to  Family suggested not to  Could not as treatment was being done  I was not present during the delivery  Other (specify)__________ | 1  2  3  4  5  6  88 |
| **If F57=1 or/and F58=1, go to F61** | | | |
| F59. | Did you want to see or hold your baby? | No  Yes, to see only  Yes, to hold only  Yes, to see and hold  Do not know | 0 1  2  3  98 |
| **If F59>0, go to F61** | | | |
| F60. | Why did you not want to see/hold your baby?  **CODE ALL THAT APPLY** | Do not want to see stillborn  Cannot bear stress  Cultural belief  Lot of paper work at facility  Other (specify)_____  Don’t know | 1  2  3  4  88  98 |

| F61. | **Interviewer: I understand it is tough as a parent to experience the loss of a child. Now, I am going to read out some statements indicating some activities that you may have done after this loss to cope with the situation. If you have done so, please say yes.  READ ALL.** | | | |
| --- | --- | --- | --- | --- |
|  |  | **Yes** | **No** | **Refused to answer** |
| A | Kept busy with work | 1 | 0 | 97 |
| B | Got involved in physical activities | 1 | 0 | 97 |
| C | Engaged in alcohol consumption | 1 | 0 | 97 |
| D | Engaged in smoking | 1 | 0 | 97 |
| E | Talked about my baby with family/friends | 1 | 0 | 97 |
| F | Expressed grief by crying | 1 | 0 | 97 |
| G | Expressed grief through aggression | 1 | 0 | 97 |

| F62. | Were you able to talk about your stillborn baby/death of your newborn baby with anyone? | No  Yes  Refused to answer | 0  1  97 |
| --- | --- | --- | --- |
| **If F62=0 or 97, go to F64** | | | |
| F63. | With whom were you able to talk about your stillborn baby/death of your newborn baby?  **CODE ALL THAT APPLY** | Wife  Mother-in-law  Mother  Father  Father-in-law  Other family member  Relatives  Friends  Doctor  FLW  Other (specify)______ | 1  2  3  4  5  6  7  8  9  10  88 |
| F64. | Did you feel that some people tried to avoid talking about the stillbirth/ death of your newborn baby when you wanted to talk about it? | No  Yes  Cannot say  Refused to answer | 0  1  95  97 |
| **If F64=0, 95 or 97, go to F66** | | | |
| F65. | Who all tried to avoid talking to you?  **CODE ALL THAT APPLY** | Wife  Mother-in-law  Mother  Father  Father-in-law  Other family member  Relatives  Friends  Doctor  FLW  Other (specify)______ | 1  2  3  4  5  6  7  8  9  10  88 |
| F66. | Did anyone avoid meeting you or seeing you post the stillbirth/ death of your newborn baby? | No  Yes  Refused to answer | 0  1  97 |
| **If F66=0 or 97, go to F68** | | | |
| F67. | Who all avoided meeting/seeing you?  **CODE ALL THAT APPLY** | Wife  Mother-in-law Mother  Father  Father-in-law  Other family member  Relatives  Friends  Doctor  FLW  Other (specify)______ | 1  2  3  4  5  6  7  8  9  10  88 |
| F68. | Did anyone pass insensitive/hurtful comments for your stillbirth/ death of your newborn baby? | No  Yes  Refused to answer | 0  1  97 |
| **If F68=0 or 97, go to F70** | | | |
| F69. | Who all passed such insensitive/ hurtful comments to you?  **CODE ALL THAT APPLY** | Wife  Mother-in-law Mother  Father  Father-in-law  Other family member  Relatives  Friends  Doctor  FLW  Other (specify)______ | 1  2  3  4  5  6  7  8  9  10  88 |
| F70. | Did anyone extend support or help to you in coping with the loss of your stillborn/newborn baby? | No  Yes  Refused to answer | 0  1  97 |
| **If F70=0 or 97, go to F72** | | | |
| F71. | Who all extended support or helped you?  **CODE ALL THAT APPLY** | Wife  Mother-in-law Mother  Father  Father-in-law  Other family member  Relatives  Friends  Doctor  FLW  Other (specify)______ | 1  2  3  4  5  6  7  8  9  10  88 |
| F72. | Did anyone blame you for this stillbirth/death of your newborn baby? | No  Yes  Refused to answer | 0  1  97 |
| **If F72=0 or 97, go to F74** | | | |
| F73. | Who all blamed you?  **CODE ALL THAT APPLY** | Wife  Mother-in-law Mother  Father  Father-in-law  Other family member  Relatives  Friends  Doctor  FLW  Other (specify)______ | 1  2  3  4  5  6  7  8  9  10  88 |
| F74. | Did anyone suggest to you to forget that there was a stillbirth/ death of your newborn baby? | No  Yes  Refused to answer | 0  1  97 |
| **If F74=0 or 97, go to F76** | | | |
| F75. | Who all suggested this to you?  **CODE ALL THAT APPLY** | Wife  Mother-in-law Mother  Father  Father-in-law  Other family member  Relatives  Friends  Doctor  FLW  Other (specify)______ | 1  2  3  4  5  6  7  8  9  10  88 |
| F76. | Were you told by anyone that mourning death of a stillborn baby/ death of a newborn baby was taboo and not culturally acceptable? | No  Yes  Refused to answer | 0  1  97 |
| **If F76=0 or 97, go to F78** | | | |
| F77. | Who all told you so?  **CODE ALL THAT APPLY** | Wife  Mother-in-law Mother  Father  Father-in-law  Other family member  Relatives  Friends  Doctor  FLW  Other (specify)______ | 1  2  3  4  5  6  7  8  9  10  88 |
| F78. | Did you want to name your baby? | Yes, named the baby  Yes, wanted to but did not  No practice of naming dead baby  Cannot say  Other (specify)_____ | 1  2  3  95  88 |

| **For ALL Fathers irrespective of any birth outcome** | | | |
| --- | --- | --- | --- |
| F79. | **Interviewer:** We would now like to ask you some questions about your mood. Please select one option for each question that is the closest to how you have felt **IN DAYS IMMEDIATELY AFTER THE DELIVERY OF [CHILD’s NAME]. READ ALL** | | |
| A | I was anxious or worried for no reason | Yes, very often  Yes, sometimes  Hardly ever  No, not at all | 1  2  3  4 |
| B | I felt scared or panicky for no good reason | Yes, very often  Yes, sometimes  Hardly ever  No, not at all | 1  2  3  4 |
| C | Things had been getting on top of me | Yes, very often  Yes, sometimes  Hardly ever  No, not at all | 1 2 3  4 |
| D | I was so unhappy that I had difficulty sleeping | Yes, very often  Yes, sometimes  Hardly ever  No, not at all | 1  2  3  4 |
| E | I felt sad or miserable | Yes, very often  Yes, sometimes  Hardly ever  No, not at all | 1  2  3  4 |
| F | I was so unhappy that I had been crying | Yes, very often  Yes, sometimes  Hardly ever  No, not at all | 1  2  3  4 |
| G | The thought of harming myself had occurred to me | Yes, very often  Yes, sometimes  Hardly ever  No, not at all | 1  2  3  4 |

| **Perception on domestic violence** | | | |
| --- | --- | --- | --- |
| **INTERVIEWER – please read this to the respondent: Domestic violence is a pattern of abusive behaviour that occurs between family members and/or intimate partners to gain power and control. Domestic violence can take the form of physical, sexual, psychological, or economic abuse**. | | | |
| F80. | I am going to ask you about some situations which happen to some women. Your answers are crucial for helping to understand the condition of women in India. Your answers will be completely confidential and will not be told to anyone in your household. If you don’t want to answer any question, just let me know and I will go to the next question.  Do you justify a husband hitting, slapping or hurting of wife if she………………….?  **READ ALL** | | |
| A | Goes out without telling him? | Yes No Don’t know  Refused to answer | 1 0 98  97 |
| B | Neglects the family or children? | Yes No Don’t know | 1 0 98 |
| C | Argues with him or other senior members in the family? | Yes No Don’t know  Refused to answer | 1 0 98  97 |
| D | Refuses to have sex with him? | Yes No Don’t know  Refused to answer | 1 0 98  97 |
| E | Doesn’t cook food properly? | Yes No Don’t know  Refused to answer | 1 0 98  97 |
| F | Suspects of her being unfaithful? | Yes No Don’t know  Refused to answer | 1 0 98  97 |
| G | Unable to conceive? | Yes No Don’t know  Refused to answer | 1 0 98  97 |
| H | Unable to give a boy child? | Yes No Don’t know  Refused to answer | 1 0 98  97 |
| F81. | For some families, the COVID-19 crisis may have increased the risk of violence. Please tell us if you have hit, slapped or hurt in other ways your wife since the lockdown started last year? **Please note that this information is completely confidential and will not be informed to anyone.** | No  Yes  Prefer not to say | 0  1  2 |
| F82. | Other than above, have you (ever) hit, slapped or hurt your wife in other ways? | No  Yes  Prefer not to say | 0  1  2 |
| F83. | **INTERVIEWER:**  Please answer this question based on the interview done with the respondent**.  DO NOT ASK THIS TO THE RESPONDENT.**    **CODE ALL THAT APPLY** | Respondents understood all the questions well  Respondent did not understand all the questions well  Respondent had difficulty in understanding the mental health questions  Respondent had difficulty in understanding the stigma related questions  Respondent was hesitant responding domestic violence questions  Respondent was very emotional during the interview  Mother/other family members were present during the interview  Other (specify)_____________ | 1  2  3  4  5  6  7  88 |

**END THE SURVEY**
